# Supplementary material for: Exploring SVA Insertion Polymorphisms in Shaping Differential Gene Expressions in the Central Nervous System
Source: Biomolecules. 2024 Mar 17;14(3):358. doi: 10.3390/biom14030358 (PMC10967846; doi:10.3390/biom14030358)
Supplement: Supplementary file 1 [file biomolecules-14-00358-s001.zip › biomolecules-2898865-supplementary.pdf]

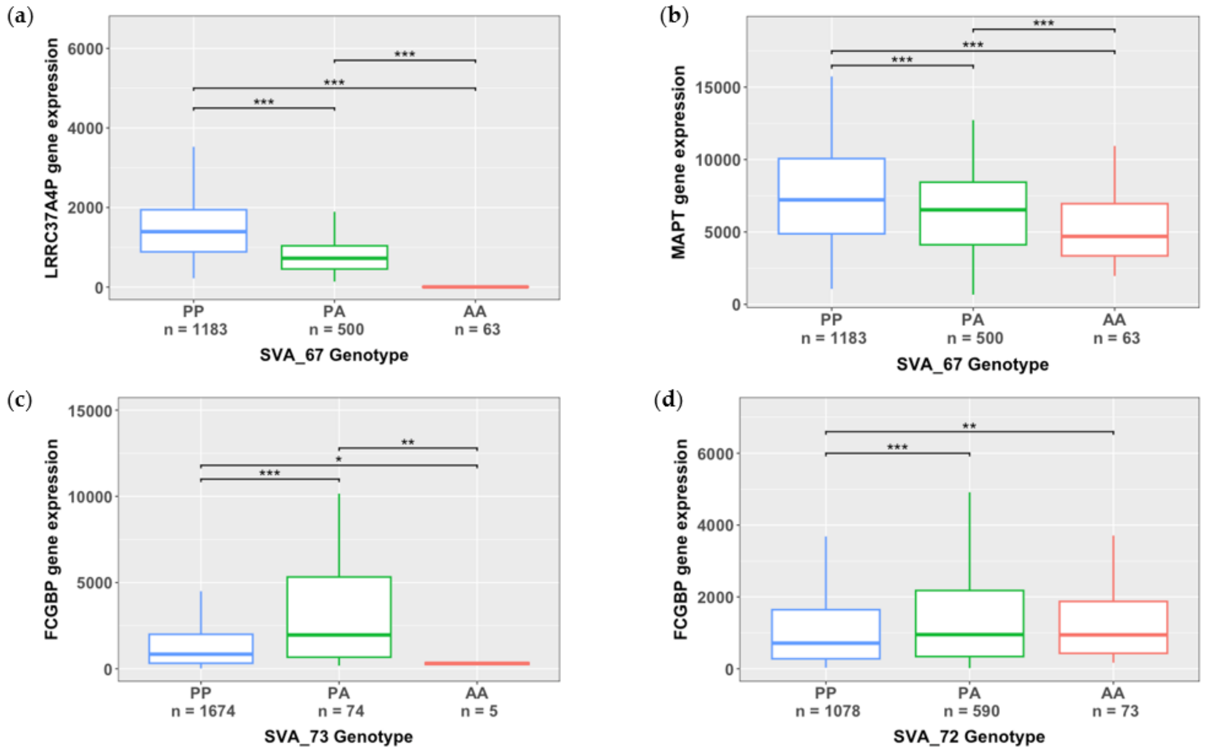

Figure S1. Boxplots of *cis*-acting SVA RIPs displaying the top two hits for largest effect size on both gene upregulation and downregulation. Datapoints from both ALS individuals and healthy controls across all CNS tissues were combined and the significance of gene expression changes between groups (*PP*, *PA*, and *AA*) was determined using the Wilcoxon pairwise comparison with FDR adjusted *p*-values (FDR<0.05). (**a**) Boxplot of *LRRC37A4P* gene expression stratified by SVA_67 genotype. Significant differences were observed between all genotype groups, displaying a 447.3-fold (*p*=1.04E-40), 214.4-fold (*p*=2.55E-38) and 2.1-fold (*p*=8.70E-77) increase in *LRRC37A4P* gene expression between the *PP* vs *AA*, *PA* vs *AA*, and *PP* vs *PA* genotype groups. Presence of SVA_67 significantly upregulated *LRRC37A4P* gene expression. (**b**) Boxplot of *MAPT* gene expression stratified by SVA_67 genotype. Significant differences were observed between all genotype groups, displaying a 1.4-fold (*p*=2.53E-09), 1.2-fold (*p*=2.51E-04) and 1.2-fold (*p*= 2.14E-12) increase in *MAPT* gene expression between the *PP* vs *AA*, *PA* vs *AA*, and *PP* vs *PA* genotype groups. Presence of SVA_67 significantly upregulated *MAPT* gene expression. (**c**) Boxplot of *FCGBP* gene expression stratified by SVA_73 genotype. Significant differences were observed between all genotype groups, displaying a 3.7-fold (*p*=3.61E-02) and 10-fold (*p*=2.61E-03) increase in *FCGBP* gene expression between the *PP* vs *AA* and *PA* vs *AA* genotype groups, whilst *FCGBP* gene expression was repressed by 63% (*p*=3.29E-08) in the *PP* vs *PA* genotype group. (**d**) Boxplot of *FCGBP* gene expression stratified by SVA_72 genotype. Significant differences were only observed between the *PP* vs *AA* and *PP* vs *PA* genotype groups demonstrating a 23% reduction (*p*=5.74E-03) and 32% (*p*=1.85E-05) reduction in *FCGBP* gene expression, respectively. A 1.2-fold (*p*= 4.23E-01) increase in *FCGBP* gene expression was indicated between the *PA* vs *AA* genotype groups, however no statistical significance was found. *p<0.05, **p<0.01, ***p<0.001.

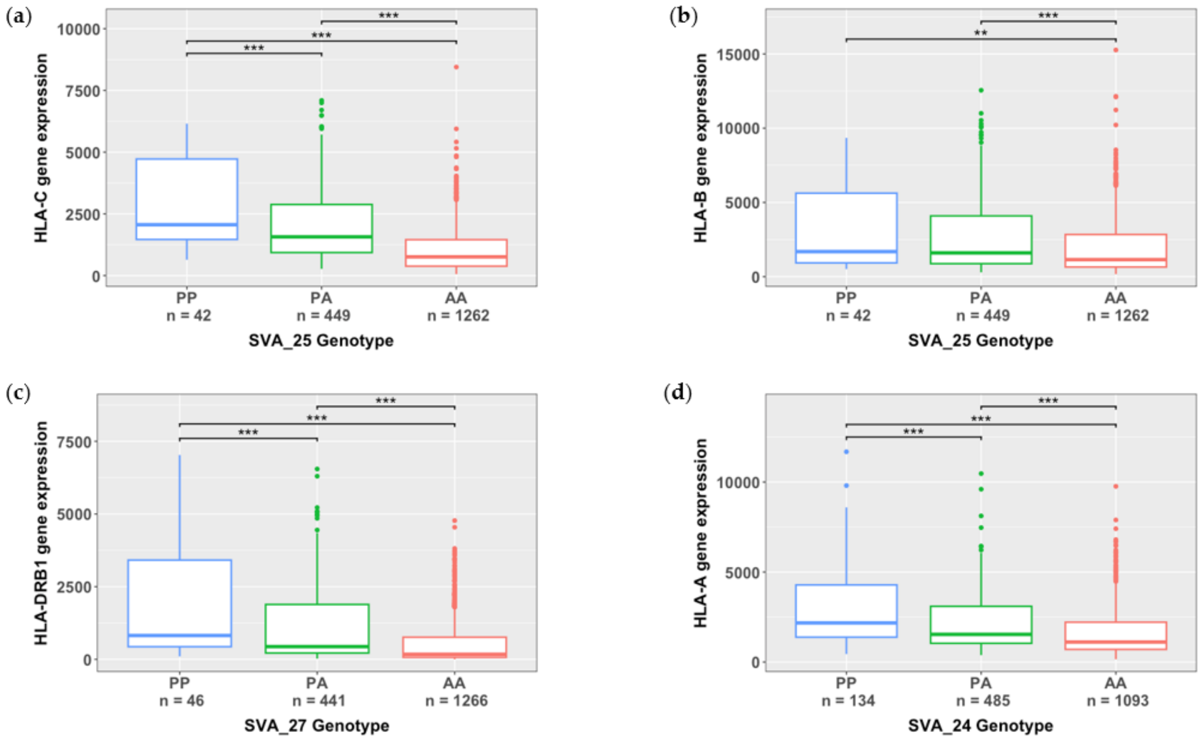

Figure S2. Boxplots of *cis*-acting SVA RIPs displaying four hits for the greatest effect size on gene upregulation. Datapoints from both ALS individuals and healthy controls across all CNS tissues were combined and the significance of gene expression changes between groups (*PP*, *PA*, and *AA*) was determined using the Wilcoxon pairwise comparison with FDR adjusted *p*-values (FDR<0.05). (**a**) Boxplot of *HLA-C* gene expression stratified by SVA_25 genotype. Significant differences were observed between all genotype groups, displaying a 3.1-fold (*p*=1.83E-13), 2.1-fold (*p*=1.82E-52) and 1.5-fold (*p*=3.18E-03) increase in *HLA-C* gene expression between the *PP* vs *AA*, *PA* vs *AA*, and *PP* vs *PA* genotype groups. Presence of SVA_25 significantly upregulated *HLA-C* gene expression. (**b**) Boxplot of *HLA-B* gene expression stratified by SVA_25 genotype. Significant differences were only observed between the *PP* vs *AA* and *PA* vs *AA* genotype groups demonstrating a 1.8-fold (*p*=2.84E-03), and 1.4-fold (*p*=1.16E-10) increase in *HLA-B* gene expression, respectively. A 1.3-fold increase in *HLA-B* gene expression was indicated between the *PP* vs *PA* genotype groups, however no statistical significance was found. (**c**) Boxplot of *HLA-DRB1* gene expression stratified by SVA_27 genotype. Significant differences were observed between all genotype groups, displaying a 5.1-fold (*p*=1.01E-10), 2.8-fold (*p*=1.84E-34) and 1.8-fold (*p*=7.26E-04) increase in *HLA-DRB1* gene expression between the *PP* vs *AA*, *PA* vs *AA*, and *PP* vs *PA* genotype groups. Presence of SVA_27 significantly upregulated *HLA-DRB1* gene expression. (**d**) Boxplot of *HLA-A* gene expression stratified by SVA_24 genotype. Significant differences were observed between all genotype groups, displaying a 1.9-fold (*p*=3.40E-18), 1.4-fold (*p*=3.40E-18) and 1.4-fold (*p*=3.44E-06) increase in *HLA-A* gene expression between the *PP* vs *AA*, *PA* vs *AA*, and *PP* vs *PA* genotype groups. Presence of SVA_24 significantly upregulated *HLA-A* gene expression. ***p*<0.01, ****p*<0.001.

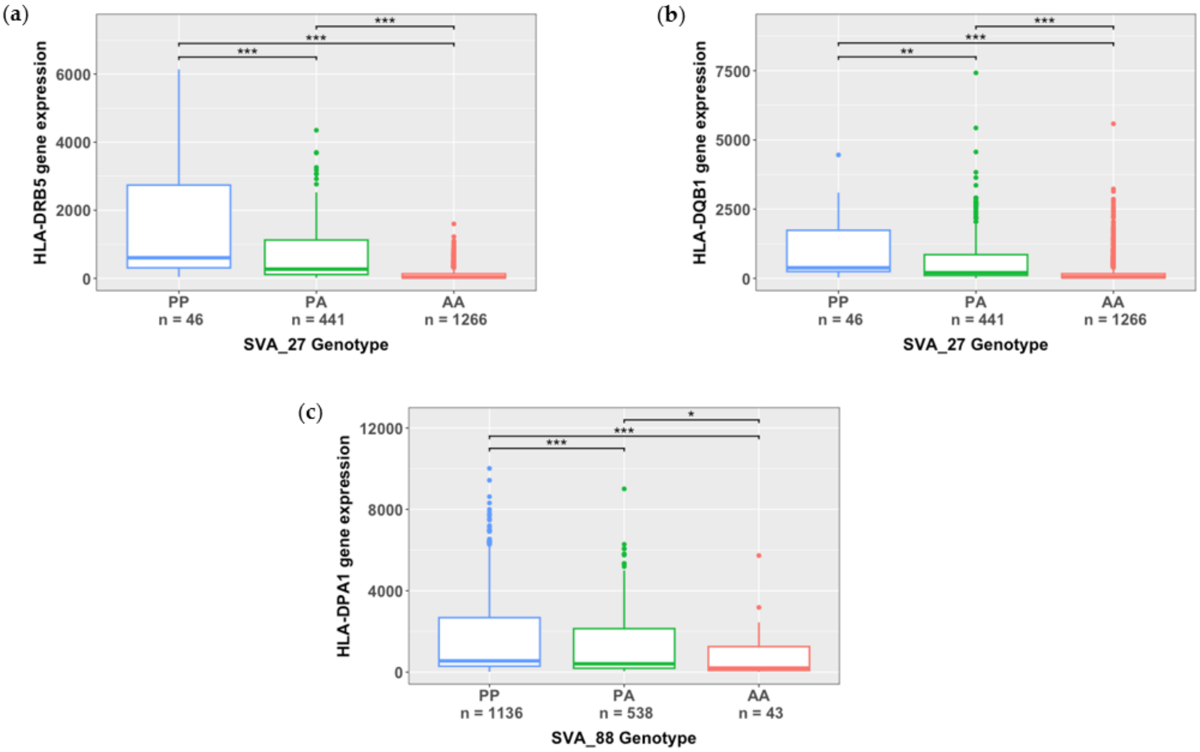

Figure S3. Boxplots of *cis*-acting SVA RIPs displaying three hits for the greatest effect size on gene upregulation. Datapoints from both ALS individuals and healthy controls across all CNS tissues were combined and the significance of gene expression changes between groups (*PP*, *PA*, and *AA*) was determined using the Wilcoxon pairwise comparison with FDR adjusted *p*-values (FDR<0.05). (**a**) Boxplot of *HLA-DRB5* gene expression stratified by SVA_27 genotype. Significant differences were observed between all genotype groups, displaying a 23.3-fold (*p*=7.51E-22), 10.4-fold (*p*=5.38E-100) and 2.3-fold (*p*=1.01E-04) increase in *HLA-DRB5* gene expression between the *PP* vs *AA*, *PA* vs *AA*, and *PP* vs *PA* genotype groups. Presence of SVA_27 significantly upregulated *HLA-DRB5* gene expression. (**b**) Boxplot of *HLA-DQB1* gene expression stratified by SVA_27 genotype. Significant differences were observed between all genotype groups, displaying a 12.4-fold (*p*=1.07E-14), 6.4-fold (*p*=1.17E-57) and 1.9-fold (*p*=2.06E-03) increase in *HLA-DQB1* gene expression between the *PP* vs *AA*, *PA* vs *AA*, and *PP* vs *PA* genotype groups. Presence of SVA_27 significantly upregulated *HLA-DQB1* gene expression. (**c**) Boxplot of *HLA-DPA1* gene expression stratified by SVA_88 genotype. Significant differences were observed between all genotype groups, displaying a 2.3-fold (*p*=7.63E-06), 1.8-fold (*p*=1.05E-03) and 1.3-fold (*p*=2.49E-07) increase in *HLA-DQB1* gene expression between the *PP* vs *AA*, *PA* vs *AA*, and *PP* vs *PA* genotype groups. Presence of SVA_88 significantly upregulated *HLA-DPA1* gene expression. *$p<0.05$, **$p<0.01$, ***$p<0.001$.
